# Mapping teachers’ awareness of artificial intelligence in the changing education paradigm: insights from a mixed methods inquiry

**DOI:** 10.3389/fpsyg.2026.1687155

**Published:** 2026-01-29

**Authors:** Ramazan Bulut

**Affiliations:** Faculty of Education, Afyon Kocatepe University, Afyonkarahisar, Türkiye

**Keywords:** AI ethics, AI risks and benefits, artificial intelligence, digitalization, education, teacher awareness

## Abstract

The proliferation of artificial intelligence (AI) tools, which reflect the rapid advancements in technology, has led to their widespread use in various fields, including health, finance and education. The effective and efficient use of AI tools in education is closely related to teachers’ awareness in this regard. The objective of this study is to comprehensively ex-amine the AI awareness status of teachers by employing a mixed-method approach. The present study is designed based on a triangulation design and is conducted with a sample of 260 teachers. The findings of the study demonstrate that teachers have a moderate level of AI awareness, and that this awareness improves through AI tool use, receiving training, following publications, and daily internet use. The qualitative findings of the study corroborate the quantitative findings in that they also demonstrate that teachers have limited awareness of conceptual perception, educational use, and positive–negative effects concerning AI tools. The findings further indicate that the majority of educators acknowledge the benefits of AI while also expressing concerns about its limitations, resulting in an ambivalent perception. The prevailing opinion among educators is that AI is not yet capable of replacing teachers due to its inability to replicate affective skills such as empathy and building rapport. However, it is also reported that the effectiveness of this phenomenon is contingent upon conscious utilization.

## 1 Introduction

Advancements in technology have given rise to a multitude of innovations that have facilitated the human condition. One of the most notable innovations is the advent of artificial intelligence (AI) technologies that emulate certain cognitive and behavioral characteristics of humans. The capacity of AI to emulate human decision-making, learning, and problem-solving skills (Triantafyllou, 2024) is a subject that can be debated and discussed. However, extending beyond these discourses, the domains in which AI technologies are employed are progressively broadening, exerting influence on numerous disparate domains. As indicated by the extant literature, AI is currently employed in a variety of fields, including education, the economy, health, transportation, communication, agriculture, autonomous systems, entertainment, military, and manufacturing (Alowais et al., 2023; Balogh, 2024; Gluoksnyte, 2025; Johnson et al., 2021; Kuhar et al., 2024; Kurtuluş, 2023; Rashid and Kausik, 2024; Salas-Pilco and Yang, 2022; Uslu, 2023; Yin et al., 2021).

The definition of AI remains nebulous. Consequently, a consensus definition remains elusive. Morandín-Ahuerma (2022) posits that AI is a machine or computer system that simulates mental processes such as reasoning, learning, and problem solving. Agrawal et al. (2023) define this technology as systems capable of performing tasks based on human intelligence and reasoning. AI has been demonstrated to facilitate a variety of cognitive processes, including prediction, association, planning, and perception (Boden, 2018). The primary objective of these technological tools is to engineer machines capable of learning, perceiving their environment, and acting autonomously to solve problems (Rashid and Kausik, 2024). Despite the heterogeneity in the definitions provided, these definitions point to a shared conception: the basis of AI is the imitation of human-specific cognitive functions by machines. One of the areas where these artificial systems have been implemented and have led to a comprehensive transformation is undoubtedly education.

The field of AI has seen significant advancements in recent years, with the development of AI models capable of emulating human cognitive functions. These developments have had a profound impact on learning environments, leading to significant changes in educational technologies. Duarte et al. (2023) posit that AI has the potential to reduce school dropout rates and enhance the teaching-learning process by providing customized and adaptable feedback to students. It has been posited that AI holds considerable potential in the development of intelligent course systems, customized learning experiences, and more effective responses to individual differences (Tilepbergenovna, 2024). Lima et al. (2024) posit that AI facilitates novel and adaptable learning styles through the personalization of instruction and the automation of text analysis. Seyrek et al. (2024) concluded that teachers indicated that AI can be utilized for material development and performance assessment. Çetin and Yıldız-Baklavacı (2024) revealed that teachers positively evaluated the use of AI and Industry 4.0 tools in education in terms of learning retention, making learning more enjoyable, providing instructional material support, and time management. According to Shribala and Jhaneswaran (2024), the utilization of AI has been shown to yield favorable educational outcomes, such as improved academic achievement and learning retention. A recent study reported that AI-supported project-based learning (PBL) models are perceived far more positively than traditional PBL approaches (Ruiz Viruel et al., 2025). As highlighted by previous research, AI offers significant potential benefits such as supporting personalized instruction, enhancing learning retention, facilitating material development, and providing effective feedback. However, its possible negative effects should also be taken into consideration.

The integration of AI into education has given rise to a number of debates. The crux of these challenges lies in the potential adverse effects and various concerns that may arise from the utilization of AI. Bu (2022) posits that the integration of AI into educational practices engenders at least three significant ethical concerns: the disruption of conventional teacher-student role dynamics, the potential compromise of educational data security, and the risk of deviating from established educational objectives. While students recognize the potential of AI to enhance their learning experiences, they have expressed significant concerns regarding privacy violations and the protection of personal data (Hasan et al., 2024). In a similar vein, Valerio’s (2024) study in Zamboanga City revealed that 55% of students harbor ethical concerns regarding job security and data privacy. A study of a sample of Pakistani and Chinese university students revealed that the most prevalent issues were inactivity (68.9%), followed by privacy and security concerns (68.6%), and decision-making challenges attributable to AI (27.7%) (Ahmad et al., 2023). Furthermore, educators have articulated concerns regarding the deleterious effects of excessive reliance on technology, particularly with respect to its impact on students’ learning motivation and critical thinking skills (Plattner et al., 2024). In addition to the aforementioned points, it is crucial to acknowledge the potential for AI systems to introduce inequalities in the data sets utilized for their training. This phenomenon, although unintended, carries the risk of generating discriminatory and unjust outcomes (Mimoudi, 2024). It is hypothesized that the mitigation of these concerns and the negative effects arising from the use of AI are contingent on educators’ awareness.

Teachers represent a pivotal demographic in the integration of AI-supported educational technologies into the education-training environment. In order to efficiently utilize the potential of AI in education and to prevent or reduce negative effects, it is imperative to map the teachers’ awareness on AI. This is due to the fact that educators function in both roles, as both consumers of AI tools and as guides. It has been posited that an increased awareness of the strengths and weaknesses of these technological tools may serve to mitigate potential risks associated with the utilization of AI, thereby facilitating its more efficient and conscientious application (Güven, 2024; Mahmood et al., 2024; Tripathi, 2024). It is also considered that this study, which addresses AI awareness encompassing dimensions such as attitude, practical knowledge, and theoretical knowledge (Ferikoğlu and Akgün, 2022), may contribute to the development of technology acceptance models. One of these theoretical approaches is the Unified Theory of Acceptance and Use of Technology (UTAUT). This theory explains technology acceptance in relation to variables such as performance expectancy, effort expectancy, facilitating conditions, gender, age, voluntariness, and experience (Venkatesh et al., 2003). The multidimensional structure of UTAUT enables technology acceptance to be evaluated in conjunction with contextual factors as well as technical competencies. However, the initial version of this theory was criticized for not incorporating other personal characteristics such as attitude, and the model was subsequently re-tested. The findings revealed that attitude has a direct effect on both behavioral intention and actual usage behavior (Dwivedi et al., 2019). In this context, it is anticipated that the findings obtained regarding awareness will contribute to the theoretical development of the dimensions of technology acceptance models, such as UTAUT, that incorporate personal characteristics. Therefore, the objective of the present study is to ascertain the extent of teachers’ awareness regarding AI. A review of previous studies reveals a paucity of research on this subject (Güneyli et al., 2024; Ünal, 2025; Uygun et al., 2024). This gap necessitates a comprehensive examination of teachers’ perceptions, knowledge, and interactions related to artificial intelligence. This study, which utilizes a mixed method approach to examine teachers’ awareness about AI, is believed to contribute to the current understanding in several ways. It reveals the current situation, identifies possible perceived risks and benefits, determines the needs for educational use of AI, and guides future AI-related education policies.

The objective of this study is to examine teachers’ AI awareness based on different types of data and to determine how this awareness is shaped by different variables. In accordance with the aforementioned objective, the present study will address the following research inquiries:

- What are the participating teachers’ AI awareness levels?
- Do teachers’ AI awareness levels differ significantly in terms of the demographical variables of gender, school type, seniority and education level?
- Do teachers’ AI awareness levels differ significantly in terms of daily internet use, the use of AI tools, receiving training on AI and reading technology-related publications?

In the qualitative part, the study also attempts to answer the following research inquiries:

- What are the participating teachers’ perceptions about AI?
- What do they know about the use of AI?
- What are their thoughts on the use of AI in the learning-teaching process?
- How do they evaluate the effects of AI on learning-teaching activities?
- What are their views on the impact of AI on the future of the teaching profession?

## 2 Method

### 2.1 Research design

The present study was conducted to examine teachers’ awareness regarding AI. To ascertain their AI awareness with greater precision and explicitness, a mixed research method is employed, entailing the collection and analysis of disparate types of data. This approach entails the collection and analysis of both qualitative and quantitative data (Creswell and Garrett, 2008). The term “mixed methods” is used to denote a variety of research designs that are employed in a single study (Greene et al., 1989; Teddlie and Tashakkori, 2015). In this study, a triangulation design was employed to enhance the validity and reliability of the findings derived from the qualitative and quantitative data, thereby facilitating the presentation of more comprehensive responses to the research inquiries. The triangulation design is a research approach in which quantitative and qualitative data on the same subject are collected and analyzed separately. The results of these analyses are then integrated to provide different yet complementary insights into the research problem. In this design, quantitative findings generally reveal overall trends and relationships among variables, whereas qualitative findings reflect individuals’ in-depth personal perspectives (Kara, 2023). The quantitative dimension of the research endeavors to elucidate teachers’ prevailing tendencies with regard to their AI awareness and its correlation with other research variables. In contrast, the qualitative dimension is designed to illuminate their appraisals of AI. The quantitative section of the study utilizes the correlational survey design, a research technique designed to ascertain the existence, direction, and strength of the relationship between multiple variables (Karasar, 2013). The phenomenology design is employed in the qualitative segment of the study. This design is a qualitative research design that aims to reveal how individuals experience a certain phenomenon from their own perspectives and how they attribute meaning to this experience (Yalçın, 2022).

### 2.2 Participants

The participants of the study are primary, secondary, and high school teachers working in the Sandıklı district of Afyonkarahisar, Türkiye. A total of 260 teachers participated in the study. Of the participants who engaged in the quantitative component of the study, 98 identified as female and 162 as male. The convenience sampling technique was employed in the study due to limitations in space, time, and economic budget that rendered the creation of a sample unfeasible (Baltacı, 2018). To collect qualitative data, 49 volunteer teachers from the same study group were included. The participant group was created using the snowball sampling technique. Both sampling strategies used in the study fall under non-probability sampling (Baltacı, 2018) and therefore carry certain methodological limitations. Convenience sampling may lead to selection bias as it relies on easily accessible participants, whereas snowball sampling may result in a more homogeneous participant group since the characteristics of the initial participants can propagate through the recruitment chain (Andrade, 2021; Gierczyk et al., 2024). Consequently, the generalizability of the findings is inherently limited, and the results should be interpreted within the contextual boundaries of the study. Nonetheless, to minimize these limitations and to ensure the inclusion of diverse perspectives, considerable effort was made to reach teachers working in different schools and across various subject areas. The participants were engaged in teaching activities in fields such as Social Studies, English, Mathematics, Primary School Teaching, Religious Culture and Moral Knowledge, Science, Information Technologies, Physical Education, Biology, Preschool Education, Turkish, Accounting and Finance, Philosophy, and Justice. The identities of the teachers were kept confidential, and each participant was represented with a code.

### 2.3 Instruments

#### 2.3.1 Artificial intelligence awareness scale

The Artificial Intelligence Awareness Scale, developed by Ferikoğlu and Akgün (2022), was employed to assess the participants’ awareness of AI. The scale employed in this study is of the 5-point Likert type. The inventory encompasses 51 items, which are organized into the following four factors: practical knowledge, belief-attitude, ability to associate, and theoretical knowledge. These sub-dimensions consist of 16 items for practical knowledge, 14 items for belief-attitude, 10 items for ability to associate, and 11 items for theoretical knowledge. The Cronbach Alpha coefficient of the scale was determined to be 0.986, and the Kaiser-Meyer-Olkin (KMO) value was found to be 0.983. The Bartlett test establishes a significance level of *p* < 0.01 (Ferikoğlu and Akgün, 2022). The minimum score that one can attain is 51, while the maximum score possible is 255.

#### 2.3.2 Artificial intelligence awareness form

An open-ended survey form was utilized to ascertain teachers’ perceptions of the concept of AI, their awareness about its general and educational use, and its effects on education and on the teaching profession. During the development process of the survey, the relevant studies were reviewed, and items were drafted. The survey form contains a total of five open-ended questions. The preliminary survey instrument was subjected to a review by an academician and a teacher with regard to its scope, linguistic usage, and expression. The open-ended questions underwent a finalization process subsequent to the implementation of modifications in terms of scope, language, and expression. These modifications were informed by the insights of subject matter experts. The items of the survey form are as follows: (1) What does artificial intelligence mean to you as a teacher? (2) What do you know about the use of AI? (3) How do you think that AI can be used in educational activities? (4) What effects do you think AI will have on educational activities? and (5) Do you think AI can replace teachers? Why or why not?

### 2.4 Data collection process and data analysis

A personal information form was utilized to collect demographic information on teachers and on study variables. An AI awareness scale was employed to ascertain the AI awareness levels of teachers. The scale was transferred to a digital environment and administered in educational institutions, including primary schools, secondary schools, and high schools. The data were collected from a total of 260 teachers who completed the survey. The normality of the data was initially verified through the utilization of descriptive statistics, visual graphics, and the Kolmogorov–Smirnov test. Given that the data satisfied the normal distribution condition and other criteria, parametric tests were employed to analyze the data (Büyüköztürk, 2012; Can, 2013). The percentage, frequency, arithmetic mean, standard deviation were used as well as t-test, one-way analysis of variance (ANOVA), and multiple comparison test used to ascertain the origin of the observed discrepancy between the groups. In addition, the total score of the Artificial Intelligence Awareness Scale and its sub-dimensions were categorized into three levels: low, moderate, and high. The cut-off points were determined by dividing the minimum and maximum score range of each sub-dimension into three equal intervals. Accordingly, the score ranges were defined as follows: for practical knowledge, 16–37 indicates low, 38–58 moderate, and 59–80 high; for belief–attitude, 14–32 indicates low, 33–51 moderate, and 52–70 high. For the ability to associate sub-dimension, 10–23 corresponds to low, 24–36 moderate, and 37–50 high; for theoretical knowledge, 11–25 represents low, 26–40 moderate, and 41–55 high levels. As for the total score of the scale, 51–119 denotes low, 120–187 moderate, and 188–255 high awareness levels. The participants’ mean scores were evaluated based on this classification.

The qualitative data were collected using an open-ended survey questionnaire. Printed copies of the form were distributed to the teachers, who were instructed to provide responses to the items. The participants were allotted sufficient time to articulate their perspectives. The open-ended survey was administered to a total of 49 teachers. The qualitative data was then subjected to content analysis. Content analysis entails the aggregation of data pertaining to specific concepts and themes, followed by its systematic organization to facilitate comprehension by the intended audience. Content analysis is a process that can be delineated into four sequential stages. As stated by Yıldırım and Şimşek (2013, pp. 259–260), these stages include the process of coding the data, identifying the themes, organizing the codes and themes, defining the findings, and interpreting the results.

The data collected from the teachers was subsequently transferred to a digital environment. Subsequently, the data were meticulously reviewed on multiple occasions prior to the initiation of the coding process. Coding can be defined as a process of inquiry that takes the form of a dialogue with the data (Merriam, 2013). Following the coding of meaningful expressions (i.e., data), the subsequent step involves the development of themes. The classification of the codes was conducted in accordance with their shared and distinct characteristics. Codes exhibiting similar features were grouped into the same category. The codes and themes obtained were then subjected to visual analysis through the use of figure and graphs, which facilitated their presentation in a systematic manner. Subsequently, the interpretation of the findings was initiated. During the presentation of the findings, direct quotations from the teachers’ opinions were used. The use of direct quotations contributes to enhancing credibility by accurately reflecting teachers’ perspectives (Tutar, 2022).

The identification of the codes and themes was facilitated by the use of a co-observer. The data were also read and coded by the co-observer, who provided support for the data analysis. During the course of the data analysis process, the research team engaged in a series of deliberations with the co-observer, with whom they convened on three separate occasions for the purpose of discussing the codes and themes. Upon completion of the process, a set of codes was established, which was met with varying degrees of acceptance and rejection. The agreement percentage between the two coders was calculated using the following formula: [Agreement / (Agreement + Disagreement) x 100]. An agreement rate that exceeds 80% is indicative of reliable coding (cited in Yapıcıoğlu, 2016: p. 76). The observed agreement rate was 88.

In this study, quantitative and qualitative data were analyzed separately within the framework of a triangulation design. In the quantitative analyses, teachers’ levels of AI awareness and the relationships among variables were identified. In the qualitative analyses, teachers’ views on the topic were presented in the form of themes and codes. After the analyses were completed, the findings were integrated in the discussion section. At this stage, the quantitative results were first addressed, and the qualitative themes were then compared with the relevant quantitative findings and interpreted. The qualitative codes were weighted according to their frequencies, and the points that supported or explained the quantitative results were highlighted. In the conclusion section, the findings obtained from both data types were evaluated together, and a holistic interpretation was provided regarding teachers’ AI awareness and its educational implications.

## 3 Results

### 3.1 Quantitative findings and their interpretation

Table 1 presents the results of the analyses regarding teachers’ levels of artificial intelligence awareness. Accordingly, based on the mean scores of the belief–attitude, ability to associate, and theoretical knowledge sub-dimensions, teachers were found to have a moderate level of awareness in these domains. In contrast, the mean score of the practical knowledge sub-dimension indicated that teachers possessed a high level of practical knowledge. Based on the total mean score, teachers’ overall artificial intelligence awareness was found to be at a moderate level. In addition, it was determined that 0.8% of the teachers demonstrated low awareness, while 56% exhibited moderate and 43% high levels of artificial intelligence awareness.

**Table 1:** Distribution of teachers’ AI awareness levels.

Sub-dimensions	*N*	$\bar{X}$	*SD*	Outcome
Belief–attitude	260	48.14	7.91	Moderate
Ability to associate	260	35.18	4.94	Moderate
Theoretical knowledge	260	40.17	5.21	Moderate
Practical knowledge	260	61.02	7.18	High
*Total score	260	184.51	20.43	Moderate

*Of the teachers, 0.8% (*n* = 2) demonstrated low, 56% (*n* = 147) moderate, and 43% (*n* = 111) high levels of artificial intelligence awareness.

As illustrated in Table 2, an independent samples t-test was conducted to ascertain whether there is a statistically significant difference in teachers’ AI awareness scores based on selected demographic and experiential variables.

**Table 2:** Comparison of AI awareness scores in terms of demographic and experiential variables using independent samples t-test.

Variables	Group	*N*	$\bar{X}$	*SD*	df	*t*	*p*
Gender	Female	98	185.77	19.34	258	0.769	0.442
	Male	162	183.75	21.08			
Use of AI tools	Yes	77	190.00	23.92	258	2.849	0.005
	No	183	182.20	18.35			
Type of schools	Public school	243	184.07	20.46	258	−1.307	0.192
	Private school	17	190.76	19.47			
AI Education	Yes	20	198.15	22.38	258	3.162	0.002
	No	240	183.38	19.88			
Reading AI publication	Yes	108	190.49	19.18	258	4.098	0.000
	No	152	180.26	20.27			

Initially, a comparison predicated on gender reveals that the mean awareness score of female participants (X̄=185.77; S = 19.34) marginally exceeds that of male participants (X̄ =183.75; S = 21.08). However, the *p*-value indicates that this difference is not statistically significant [t (258) = 0.769; *p* > 0.05]. Consequently, it can be concluded that gender does not significantly influence participants’ AI awareness levels. The findings indicate a significant discrepancy between the groups with respect to the utilization of AI tools. Participants who reported using AI tools exhibited a higher mean awareness score (X̄ =190.00; S = 23.92) compared to those who had not (X̄ =182.20; S = 18.35), and this discrepancy was statistically significant [t (258) = 2.849; *p* < 0.01]. This finding suggests that experience with AI tools is associated with increased awareness.

With respect to school type, teachers working at private schools (X̄=190.76; S = 19.47) demonstrated higher mean scores than those working at public schools (X̄=184.07; S = 20.46). However, the observed difference was not statistically significant [t (258) = −1.307; *p* > 0.05].

A notable discrepancy was observed in relation to the participants’ training background in AI. The participants who had undergone such training exhibited significantly higher levels of awareness (X̄=198.15; S = 22.38) in comparison to those who had not received training (X̄=183.38; S = 19.88) [t (258) = 3.162; *p* < 0.01]. This finding suggests that AI education contributes meaningfully to increased awareness.

The data demonstrates a significant discrepancy in awareness scores contingent on participants’ engagement with technology-related publications. Participants who reported reading such materials (X̄=190.49; S = 19.18) exhibited significantly higher scores compared to those who did not (X̄=180.26; S = 20.27) [t (258) = 4.098; *p* < 0.001]. This finding suggests that perusing contemporary publications on AI, in both printed and digital formats, contributes to heightened awareness levels.

Table 3 presents the one-way ANOVA results comparing teachers’ AI awareness scores across daily internet use, seniority and school level. A significant difference was identified only for daily internet use (*F* = 3.11, *p* = 0.016). Multiple comparison results show that teachers who use the internet for 5–7 h per day have significantly higher AI awareness scores than those who use it for less than 1 h. In addition, teachers who use the internet for more than 7 h daily scored significantly higher than teachers who use it for less than 1 h, 1–3 h and 3–5 h. Therefore, it is plausible to hypothesize that the participants’ daily internet use exerts an influence on their AI awareness scores. It can be posited that groups with relatively high daily internet usage time exhibit significantly higher AI awareness scores.

**Table 3:** Summary of ANOVA results on AI awareness scores by daily internet use, seniority and education level.

Variable	Source of variance	Sum of squares	df	Mean of square	F	*p*	Significant difference
Daily internet use	Intragroup	4566.61	4	1141.65	3.11	0.016	4–1, 5–1,* 5–2, 5–3
	Intergroup	91865.94	250	367.46			
	Total	96432.55	254				
Seniority	Intragroup	1317.11	5	263.42	0.63	0.679	—
	Intergroup	106733.85	254	420.21			
	Total	108050.97	259				
Education level	Intragroup	544.28	2	272.14	0.65	0.522	—
	Intergroup	104735.65	251	417.27			
	Total	105279.93	253				

*Groups: 1 = less than 1 h, 2 = 1–3 h, 3 = 3–5 h, 4 = 5–7 h, 5 = more than 7 h of daily internet use.

In contrast, the ANOVA results for seniority indicated no significant differences in AI awareness based on years of teaching experience. This suggests that seniority does not have a meaningful impact on teachers’ AI awareness levels. Similarly, the analysis based on the education level (primary, secondary, and high school) revealed no significant variation in awareness scores across different school levels. This finding indicates that the type of school at which teachers are employed does not substantially influence their AI awareness.

### 3.2 Qualitative findings and their interpretation

The first inquiry posed to the participants in the interview form pertained to: “What does artificial intelligence mean to you as a teacher?” Forty-eight out of forty-nine participants provided a response to this question, while one participant stated that s/he had no opinion. The analysis yielded two overarching themes: definitions and views.

As shown in Supplementary Figure 1, the definitions theme consists of seven codes. The views theme is divided into two subthemes, namely positive and negative views. The initial code of the definitions theme pertains to technology that facilitates daily living. Thirteen of the teachers defined AI as technological developments that facilitate human life in various domains. For instance, the participant 34 defines AI as follows: “A tool that makes people’s lives easier and helps solve some complex tasks.” Another code obtained from participant views is machine learning. A total of thirteen participants defined AI as machine learning. This phenomenon is elucidated in the following statement by participant 32: “Technology has gained the ability to think and make its own decisions.” Participant 18, one of the ten participants who expressed their views on AI as systems that imitate humans, describes AI as follows: “It is a system that imitates human intelligence to perform certain tasks.” Another component of this theme is an educational tool. A total of ten participants defined AI as an educational tool. For instance, participant 41 articulated his perspective as such: “I think it is an effective technology that can be used when producing activities and materials for different student profiles and students with different learning styles.” Another code that emerged in the definitions theme is “risky tool,” which occurs four times. In their definitions, the participants placed particular emphasis on the potential risks associated with AI. The following are the views of participant 4: “I think artificial intelligence is both indispensable for the new age and a scary situation.” Three participants provided views on the code of AI as a data analysis and evaluation method. One of them, participant 14, expressed his views as follows: “I call AI as the transferring of collected data to digital environment and obtaining stronger probabilities to be evaluated.” One of the six participants who regarded AI as the automation feature code, participant 47, expressed his views as follows: “When it comes to AI, the first thing that comes to my mind is the automation of tasks or processes that can be done by human power by computers.”

As illustrated in Supplementary Figure 1, the theme of positive views is comprised of two distinct codes: general positive views and education-related positive views. Within the domain of negative perspectives, three distinct codes have been identified: economic risks, general fear, and educational risks.

A set of three codes was developed in the theme of negative views. The participants’ negative views about AI are associated with economic and educational risks, while others are rooted in general fears for present or future activities. The following are the views of participant 16, one of the three participants who drew attention to the dangers of AI in the economic field: “I think that betting web-sites, crypto markets and those who use AI in the stock market are useful for defrauding citizens.” A subsequent analysis revealed that four participants articulated their sentiments within the overarching category of fear. Participant 2 articulated his perspective as such: “I think there is no limit to what they can do and I am very afraid of the fact that humans can misuse it.” These reports indicate that some participants have expressed concerns regarding the utilization of AI. The final code in this series pertains to the subject of educational risks. The sole participant who has articulated an opinion on this issue has asserted that the integration of AI in educational settings has the potential to engender a culture of complacency among students, thereby stifling their creative potential.

The following sentences present the positive views of participant 33: “It can be really useful especially when time is limited.” Participant 35 expressed a favorable opinion of AI in the context of education: “… It allows students to spend more time and increases the transition from abstract to concrete in teaching.”

Teachers define AI as technological developments that facilitate work in different areas of life, machine learning, systems that imitate humans, educational tools, risky tools, tools that can analyze and evaluate data, and systems that provide automation. Conversely, a minority of teachers delineate AI as a tool that carries risk due to the potential risks that may arise from its uncontrolled use. Additionally, six teachers opted to address the positive or negative consequences arising from the use of AI, eschewing a definition or explanation of the concept. One teacher has indicated that he lacks familiarity with this concept. Rather than defining the concept of AI, the teachers briefly discuss the positive and negative consequences of its use, indicating that their AI awareness at the conceptual level is inadequate.

The second question asked to the participants was: “What do you know about the use of AI?” The responses to this inquiry enumerate nine distinct areas of utilization, with all participants offering their perspectives on the subject. As illustrated in Supplementary Figure 2, the aforementioned areas encompass economic, healthcare, education, communication, security, transportation, arts, technology, and social domains.

It is reported by participants 1, 12, 16, 33, 43, and 44 that the application of AI may be applicable to all fields of study. The field of economics has received the most citations in the context of AI. In this particular domain, participant 1 articulates the ensuing perspective: “…I think AI will be used in factories, workplaces, and everywhere where people work in the future.” The field of healthcare emerges as the second most frequently cited domain of AI implementation among the study’s participants. Participant 34 articulates his perspective on the subject in the following manner: “I know that it is used especially in healthcare services in areas such as diagnosis and treatment, x-ray, MRI, patient monitoring.” A total of fourteen participants have indicated their belief that AI will be utilized within the educational sector. In regard to this matter, participant 14 offers the following perspective: “In the field of education, it is used in student achievement monitoring, educational coaching, evaluation scales, and question pools, which have just begun to emerge.”

Twelve participants in the study indicate that AI is employed in the context of communication. For instance, participant 36 reports the following: “With artificial intelligence translation programs, the process (communication) can be facilitated without any communication problems with the other person.” The participants’ responses in the domain of security indicate that concepts such as bomb disposal, facial recognition in law enforcement, unmanned aerial vehicles (UAVs), UAV production, cyber security, social security, and defense industry are encountered. A total of ten participants incorporated the domain of security within their respective responses. One of the participants, number 18, has provided the following perspective: “It is also used in sector-specific strategic studies such as cyber security and defense industry.” Transportation emerges as a domain of AI utilization, with eight participants incorporating it within their responses. The concepts of autonomous driving, logistics, navigation, traffic flow, and airport usage have emerged in the field of transportation. Participant number 34 articulated the following perspective on the subject: “It is used in the fields of transportation and logistics.”

Art is the field that seven participants include in their answers. Examples of AI’s use in the field include photo shooting, Photoshop design applications, photo and video designs, song editing, painting technology, and cinema. Participant 48 articulated his perspective as such: “It is used in many areas from painting to making music.” According to six respondents in the study, AI has the potential to be applied in the field of technology, which was identified as one area where such technology could be implemented. Participant 31 provided the following perspective: “In technological fields.” Four participants state that AI can be employed in social life. Participant 30 stated they are employed “in smart homes.” Another participant answer referred to “daily life.”

Upon evaluation of the participant’s responses to the second question, it can be concluded that they possess a range of information regarding the various domains of application of AI. A select group of participants, numbering six, adopt a more expansive stance on the matter, asserting that AI has the potential to be applied across various disciplines. The utilization of general expressions by these teachers indicates a lack of consensus among participants regarding the domains of AI implementation. In addition to these resources, other faculty members offer more detailed explanations about the specific applications of AI. In the responses provided by the aforementioned teachers, the subjects of economy, health, education, and communication were frequently prioritized. In addition to these domains, the report also encompasses subjects such as security, transportation, art, technology, and social life.

The third question asked to the participants was: “How do you think AI can be used in educational activities?” The results of the analytical process undertaken in response to this inquiry, in which forty-nine subjects were invited to share their perspectives, yielded the codes illustrated in Supplementary Figure 3.

As illustrated in Supplementary Figure 3, the individualization of education, as articulated by fifteen participants, is regarded as one of the most advantageous attributes of AI in educational settings and learning activities. Participant 34 advances the following perspective: “Each student’s intelligence level, interests and abilities are different from each other. At this point, AI can individualize education according to everyone’s level and abilities and can provide the students with an opportunity to learn at their own pace.” Thirteen participants make reference to the implementation of AI in measurement and assessment. The following observations are attributable to participant 37: “At the end of the first and second exams prepared with the support of AI at secondary schools, high schools and universities, individual deficiencies of the students can be detected in line with the exam analyses to be made by AI systems and individualized program application can be provided.” Eleven participants have attested to the efficacy of AI in facilitating teaching and learning. Participant 28 expounds on the following perspective: “It makes learning a subject easier, enables the determination of the most appropriate method and technique for learning.” Participant 38 expresses the following: “AI can be used for more efficient teaching.” Ten participants state that AI would contribute to concretization. Participant 36 expresses the following opinion on the subject: “A more dynamic lesson environment can be created by designing presentations, videos and visuals for the lesson.” On the same topic, participant 35 states the following: “AI is beneficial in concretizing abstract information.” Participant 47, one of the seven participants who asserted that AI would contribute to material development, provided the following statement: “It may be effective in providing proper education materials to students.” It has been posited by certain participants that the advent of AI may facilitate convenient access to information. One of the six participants who has expressed this perspective is participant 2, whose views are as follows: “Educators, teachers, and students can access the information they want by filtering the topic they want to research and learn about.”

There are four participants who argue that AI may help teachers in the education-learning process. One of them is participant 18 who reports the following: “Artificial intelligence can identify individual learning needs of students and guide teachers.” Participant 24, one of the three participants who stated that AI would be used in the curriculum development process, expressed the following: “AI may detect learning deficiencies and may develop a proper curriculum for students.” With regard to the subject of content creation via AI, previously mentioned by two of the participants, participant 42 makes the following assertion: “Content can be developed through AI based on children’s individual differences.” Two participants expressed their views on creating classroom order through AI. Participant 8 expresses the following view on this subject: “It can be used in organizing the classroom environment.” Finally, two participants offer their perspectives on the potential of AI to facilitate perpetual learning. Specifically, participant 13 asserts that AI has the capacity to enable permanent learning, with the following statement: “Instead of historical field trips, more permanent learning can be achieved with 3D simulations.”

The prevalence of issues such as individualization of education, effective teaching-learning methodologies, measurement-evaluation techniques, concretization, and material development indicates that educators hold a favorable perception of the potential of AI to enhance educational functionality. Conversely, the infrequency with which teachers address certain areas of use, such as administrative functions (e.g., curriculum and content preparation, assistance to teachers, and the establishment of classroom order), and the superficial nature of their responses, suggests a limited awareness of these issues. In consideration of the aforementioned findings, it can be posited that their cognizance of AI implementation in domains such as planning and educational administration is suboptimal.

The fourth question asked to the participants is as follows: “What effects do you think AI will have on educational activities?” Four of the teachers chose not to respond to this question. Participant 4 stated: “It is too early to say something about it.” A total of forty-four participants provided their perspectives on this question. Of these participants, 32 (73%) acknowledged that AI possesses both positive and negative dimensions. Seven participants (16%) emphasized only positive aspects of AI, while five participants (11%) focused solely on its negative aspects.

The participants’ views regarding the effects of AI use were classified under positive and negative themes. All codes related to these two themes are presented in Supplementary Figure 4. As shown in the figure, the most prevalent concern expressed by the participants is the tendency of AI to cause individuals to become lazy. Thirteen participants have expressed the opinion that AI has a detrimental effect on human motivation, leading to a decline in physical and mental activity. Participant 37 offered the following perspective on the matter: “When students want many things to be done by AI, it can cause laziness.” Nine participants have reported that AI has the potential to diminish research and cognitive abilities. In relation to the aforementioned subject, participant 31 articulates the ensuing perspective: “I think it will have more of a negative impact. Today’s youth continue their lives completely addicted to technology. With AI, they can kill their creativity by doing homework and operations with AI, without doing any research or preparation.”

As posited by seven participants, an additional adverse aspect of AI is that it encourages individuals to exploit it. With regard to the aforementioned issue, participant 6 proffers the following perspective: “It can be negative because students may look for something ready while doing homework.” Seven participants indicated that the demerit of AI lies in its inability to establish a social–emotional bond. In this particular instance, participant 3 endeavors to draw attention to the fact that social connections cannot be established between students and AI by means of the following assertion: “With artificial intelligence, it will not be possible to take into account that humans are an emotional, social and physical whole in education-training activities and there will be no student-teacher interaction.” Seven participants have expressed concerns regarding potential privacy and security issues. Participant 34 further elaborates on this perspective, articulating the following viewpoint on the matter: “Privacy and security issues may arise with the improper use of AI. Data gaps in the system where students’ information is processed put student information at risk.”

Five participants have indicated that AI has the potential to induce dependency. With respect to the aforementioned topic, participant 24 offers the following contribution: “Students are already addicted to screens, phones and computers, and with such applications, it becomes easier for them to become addicted to screens, phones and computers.” Four participants have indicated that AI has the potential to give rise to ethical concerns. Participant 11 offers the following commentary on the issue: “… it can negatively affect their morality and development and present them with content that is not appropriate for their age.” Three participants have indicated that the accessibility of AI is a potential concern, as its availability may not be uniform across all demographics. This scenario could lead to the emergence of disparities due to variations in access and utilization. Participant 15, for instance, advances the following argument: “There may be injustice among students, not everyone can access it because it is an expensive product.”

Two participants have indicated that the advent of AI may result in a significant increase in unemployment. In addition to the aforementioned aspects, the participants reported the following negative elements: The potential implications of AI in this context are numerous and pervasive, including the capacity to induce distraction, the potential for deficiencies in needs analysis, the possibility of diminishing motivation, the propensity to engender psychological distress, the likelihood of exerting a deleterious effect on academic achievement, and the capacity to diminish reading habits.

According to Supplementary Figure 4, the foremost positive feature of AI is its role in enhancing access to information. The most salient benefit of AI is its capacity to facilitate access to information. An exemplification of said code, as referenced by nine participants, is illustrated by participant 3’s perspective: “Students will have easy access to the source of information and learning will continue in every environment.” Eight participants have articulated their perspectives on the potential of AI to enhance learning environments. Participant 6 offers the following perspective on the subject: “It enriches the learning environment and makes it productive.” Participant 13 states: “A war to be told can be transformed into a simulation where the person will be in that war with a software and graphic support produced by artificial intelligence.” arguing that learning environments may become enriched and productive through AI. As indicated by the responses of seven participants, the potential for AI to expedite tasks and conserve time was a salient point of discussion. Participant 29 articulates the following opinions on the subject: “Students can find answers to the questions they want to learn and seek answers to very quickly with programs created with artificial intelligence.”

The role of AI in individualized education is a topic that is addressed by four participants. Participant 37, one of the four participants, states the following in relation to this matter: “Since education and training activities will be supported by AI, these systems can provide the course flow according to the topics the student needs or is deficient in.” The issue of material development is included in the opinions of three different participants. Participant 47 expresses the following view on this subject: “I think it will be effective in providing students with appropriate educational materials.” Furthermore, three participants have expressed their conviction that AI has the potential to assist teachers. The following presents the opinions of participant 35 on the aforementioned subject: “I think it can be useful in making the teacher’s job easier.”

In addition to the aforementioned points, the provision of ongoing learning opportunities, the capacity to attract attention, the facilitation of experimentation, the maintenance of impartiality, the reduction of costs, the enhancement of institutional performance and productivity, the generation of novel production concepts, and the promotion of multidimensional thinking have been identified as positive aspects. A substantial proportion of the participants have indicated that AI is accompanied by both advantages and disadvantages. A select few participants offered a limited assessment, mentioning only the positive or negative aspects. This finding suggests a notable lack of awareness among participants regarding the potential risks and benefits associated with the educational implications of AI.

Finally, the participants were asked the question “Do you think AI can replace teachers? Why or why not?” Forty-six participants provided responses to this question, while three did not respond. As illustrated in Supplementary Figure 5, 31 of the respondents indicated that AI is not a substitute for the teaching profession, while 9 respondents expressed the contrary view. Six of the participants reported that it could partially replace it.

The prevailing sentiment among teachers is that AI will not supplant their roles in the foreseeable future. According to 20 out of 31 participants who hold this perspective, the primary rationale for this phenomenon is attributed to the perceived absence of emotions in AI. For the aforementioned participants, who posit that education encompasses not merely the transmission of information but also the emotional dimension inherent in the learning-teaching process, AI is deemed inadequate as a substitute for educators. Participant 16 eloquently articulates the following: “I do not think AI can replace a human teacher. Also, a human is not just a body like a robot, it has emotions. Particularly at primary school, children want to hug, touch, feel safe. It may be better than a teacher in terms of knowledge, but it cannot replace a teacher.”

Another rationale posited for the irrelevance of AI in replacing teachers is its capacity to merely supplement their efforts. Nine participants advocate for the position that AI is not intended to supplant teachers; rather, it is designed to augment their efforts. Participant 17 offers the following statement on the matter: “I think artificial intelligence can contribute to our profession by collaborating with us. It is out of the question for it to replace teachers.” The notion that AI is incapable of supplanting educators is predicated on the premise that AI is a human creation. This perspective was articulated by three participants in the study. Participant 4 shares the following views on this issue: “I do not think that anything artificial can replace humans. Humans are already created flawlessly on their own, while AI is a human product.” In addition to these, a multitude of factors have been posited as justification for the conclusion that AI is not a suitable replacement for educators. Among these factors are the following: the requirement of a robust infrastructure; the inability to facilitate socialization; and its limited capacity to transmit values.

Conversely, four out of nine participants who advocate for the notion that AI could substitute for educators also provide the rationale behind their perspective. For instance, participant 19 reports the following: “People are moving toward robotization.” Participant 28 gives the following reason: “Fast developments in technology.” Participant 39 expresses the following: “It would be much more helpful in many cases.” Participant 45 argues the following: “The speed of artificial intelligence.” The other participants who defend this view did not provide a rationale for their ideas. Participants 33 and 48 posit that, while the present moment may not be conducive to the replacement of teachers by AI, such a prospect may become more realistic in the future.

A total of six participants advanced the argument that AI has the potential to substitute, at least in part, for teachers in the classroom. Participant 14 makes the following statement: “AI will replace teachers in a limited way. The mental, physical and emotional development of students starting from primary school will be monitored with AI and possible disruptions that may occur in the future will be determined in advance.”

A general evaluation of the participants’ views reveals that more than half of them stated that AI cannot replace the teaching profession for various reasons. Furthermore, these participants provided justifications for their perspectives. A number of participants have expressed the view that the teaching profession cannot be entirely or partially replaced by AI. Teachers who expressed the view that AI can supplant educators either encountered challenges in articulating the rationales underpinning these perspectives or did not provide any rationale at all.

## 4 Discussion, conclusion and recommendations

In this study, teachers’ awareness of artificial intelligence (AI) was examined using a triangulation design. This design offers the opportunity to explore the research topic in a more in-depth and multidimensional manner. In the quantitative analysis, teachers’ levels of AI awareness and their relationships with various research variables were investigated. In the qualitative analysis, participants’ conceptual perceptions of AI, their views on its areas of use, and their evaluations regarding its impact on the teaching profession were explored. The qualitative codes were analyzed based on their frequencies, and statements that supported, explained, or provided alternative perspectives to the quantitative findings were especially highlighted. While the quantitative data revealed general trends, the qualitative data provided a more detailed and explanatory perspective on the same topic. By interpreting both data types together, a multifaceted and comprehensive understanding of teachers’ AI awareness was achieved.

The quantitative findings indicate that teachers’ overall artificial intelligence awareness is at a moderate level. This finding is consistent with previous studies showing that the artificial intelligence awareness levels of teachers and pre-service teachers are at a moderate level (Gaber et al., 2023; Parthiban and Ganesh, 2024; Uygun et al., 2024). However, when the sub-dimensions of awareness are taken into account, a more detailed awareness profile emerges. Teachers’ high level of awareness in the practical knowledge sub-dimension indicates that they have knowledge about the technical functioning of AI, its data-based structure, and some of its application areas. On the other hand, it was observed that awareness levels in the belief–attitude, ability to associate, and theoretical knowledge sub-dimensions were at a moderate level. This indicates that teachers have not fully deepened their belief–attitude regarding the role of artificial intelligence in education, their ability to relate this technology to different contexts, and their level of knowledge about its theoretical foundations.

The fact that artificial intelligence awareness is generally at a moderate level shows that certain forms of awareness about artificial intelligence, which has relatively recently entered the field of education, have begun to develop. From a pedagogical standpoint, this indicates that teachers are aware that artificial intelligence is meaningful for educational practices. However, they have not yet reached a level of maturity that would allow them to transform this awareness into comprehensive instructional design and classroom practice. In this context, the limited level of awareness in the belief–attitude, theoretical knowledge, and ability to associate sub-dimensions can be interpreted within the UTAUT framework. According to this theory, technology acceptance is shaped by performance expectancy, effort expectancy, social influence, facilitating conditions, and other moderating variables (Venkatesh et al., 2003). Inadequate technological infrastructure in schools, limited artificial intelligence–oriented professional development opportunities for teachers, and the lack of institutional policies that support the use of artificial intelligence may weaken facilitating conditions. It can be stated that this situation limits teachers’ transformation of their high level of awareness in the practical knowledge sub-dimension into a more holistic, critical, and safe use of artificial intelligence. These results indicate that professional development programs aimed at deepening teachers’ attitudinal, contextual, and theoretical awareness of artificial intelligence are necessary. If explicit institutional support and clear guidelines for the use of AI in education are not provided, it will be difficult to enhance the current level of awareness and to translate it into responsible adoption.

The study’s findings reveal that there is no statistically significant difference in AI awareness based on gender, suggesting that teachers’ gender does not influence their perception of AI. A number of studies have been conducted with both pre- and in-service teachers. These studies have reported that gender does not have a significant effect on AI awareness (Dandinker, 2025; Parthiban and Ganesh, 2024; Uzun, 2025). As indicated in the study by Güneyli et al. (2024), which was conducted on a sample of teachers, the results indicate that there is no discrepancy in AI awareness based on gender. Conversely, some studies have demonstrated a substantial impact of gender on AI awareness and utilization (İçöz and İçöz, 2024; Rajki et al., 2025). In the study by Đerić et al. (2025), which was conducted on 883 students, educators, and researchers working at a university in Croatia, it was found that women demonstrated higher AI awareness. Conversely, studies examining the AI awareness of pre-service teachers and healthcare professionals have reported that males exhibit higher levels of AI awareness (Çağlar, 2024; İçöz and İçöz, 2024). On the other hand, it has been observed that gender does not exert a substantial influence on AI awareness in certain studies. However, in other studies examining the relationship between gender and AI, gender has been found to be a determining factor in this regard. Consequently, a consensus on this subject has yet to be reached. The absence of a discernible impact of gender in the present study may be attributed to the contemporary exposure of both men and women to analogous digital tools. For instance, studies have shown that both men and women possess comparable levels of digital literacy (Aksoy et al., 2021; Bay, 2021; Kozan and Bulut Özek, 2019; Sarıkaya, 2019). It is hypothesized that, given the potential parity in digital literacy between male and female teachers, their cognizance of AI, a field intimately associated with digital technologies, may also be comparable.

The current study’s findings indicated a significant relationship between teachers’ daily internet use and their AI awareness levels. However, the findings of the study conducted by Banaz and Demirel (2024) on pre-service teachers suggest that daily internet use exerts no influence on their AI literacy. These contradictory findings suggest that daily internet use alone cannot always increase AI awareness, and that the reason for users’ internet use should also be taken into consideration. The participants of the aforementioned study, which found no significant effect of daily internet use on AI literacy, were pre-service teachers. In contrast, the participants of this study are in-service teachers. This discrepancy may potentially modify the objectives associated with internet utilization. A significant discrepancy has been identified between teachers who utilize the internet for over 5 h daily and those who utilize it for less time. The former group exhibits a notably higher level of AI awareness. Teachers who spend more time on the internet are more likely to encounter AI-related content through social media and news sources. This exposure may provide them with greater opportunities to acquire information about AI, thereby granting an advantage in terms of awareness. Furthermore, the absence of in-service training on AI for teachers, or the inadequacy of existing training, underscores the pivotal role of the internet in enhancing AI awareness. Consequently, it appears unavoidable that educators who have been utilizing the internet for an extended period will possess a high degree of AI awareness. It is imperative to consider the positive impact of the internet, despite the fact that there are individuals who use it in an addictive manner. As asserted by Aleebrahim et al. (2022), spending excessive amounts of time on the internet can result in internet addiction, which can have a variety of detrimental consequences. Consequently, the execution of mixed-method studies is imperative to elucidate the intricate relationship between internet addiction and AI awareness with greater precision.

The findings of the study demonstrate that teachers who utilize AI tools exhibit higher levels of AI awareness in comparison to those who do not employ such tools. This phenomenon can be attributed to the individual’s familiarity with technological tools. The findings suggest that experience with AI tools leads to an enhancement in AI awareness levels. This result aligns with prior research findings, thereby supporting the robustness of the study’s conclusions. As indicated in the study conducted by Güneyli et al. (2024), knowledge regarding the applications of AI fosters a more pronounced level of AI awareness in comparison to the possession of solely theoretical knowledge. Uzun's (2025) study posits that AI awareness levels are significantly higher among those whose technology use exceeds three hours. Ünal (2025) further posits that, within the application knowledge sub-dimension, teachers using technology for over four hours show higher AI awareness compared to those who engage with technology for less than this duration. In light of these findings, it can be posited that teachers’ learning AI through experiential learning has a substantial impact on their AI awareness. Consequently, it is proposed that in-service training programs should incorporate not only the theoretical underpinnings of the educational application of AI but also opportunities for teachers to engage in hands-on practice.

The incorporation of AI applications training prior to in-class utilization has been demonstrated to mitigate technostress. Research indicates that educators experience elevated levels of stress when confronted with technological tools that do not function as intended. Furthermore, factors such as the necessity to adapt to technology, the inherent complexity, uncertainty, and insecurity of technology have been identified as stress-inducing elements (Bourlakis et al., 2023). Additionally, it has been observed that the integration of novel technological devices into the educational environment can potentially induce technostress among teaching professionals (Khlaif et al., 2023). The pressure experienced by teachers can be mitigated through the provision of training in applied AI, which may encompass digital literacy skills. Indeed, a number of studies have indicated that digital literacy and technopedagogical content knowledge are inversely related to technostress. Furthermore, digital competencies have been demonstrated to be a contributing factor in the alleviation of technostress (Bartra-Rivero et al., 2024; Gökbulut, 2021; Muslimin et al., 2023; Özgür, 2020; Vásquez-Pajuelo et al., 2024).

This study revealed that working at a public or private school does not significantly differentiate teachers’ AI awareness. This finding is corroborated by a study examining teachers’ AI awareness, which concludes that working at a private or public institution does not significantly affect AI awareness. The underlying rationale for this phenomenon may be associated with individual qualifications or educational policies rather than the institution to which educators are affiliated (Güneyli et al., 2024). It can be argued that this similarity stems from the fact that neither type of institution has yet integrated AI-related training or AI-based instructional practices sufficiently into their educational processes. This is particularly salient in institutions that have yet to incorporate AI-related components into their educational processes. The integration of AI technologies into educational settings necessitates the implementation of specialized technical infrastructure, including a robust internet connection and hardware and software components, which collectively incur higher expenses. The dearth of adequate resources for technological infrastructures within educational institutions has the potential to impede the development of teachers’ knowledge and skills regarding AI. It has been hypothesized that this situation does not engender a substantial discrepancy in the awareness of teachers working at public and private institutions that compete in the education sector.

The investigation revealed no statistically significant correlation between teachers’ seniority and their AI awareness levels. This result aligns with previous findings in the field. For instance, Ünal (2025) posits in his study that teachers’ professional seniority and age do not have a significant effect on their AI awareness. A study on the use of AI for educational purposes found that professional teaching experience does not influence perceptions of AI (Lawal et al., 2025). Güneyli et al. (2024) found that teachers’ age does not have a significant impact on their AI awareness. However, it should be noted that other studies have yielded divergent results. In the study conducted by Uygun et al. (2024), it was determined that professional teaching experience exerts no significant influence on total AI awareness scores. However, a notable disparity was observed in the practical knowledge sub-dimension, with younger teachers demonstrating higher scores in this regard. The same study also reported that age has a significant effect on the AI awareness of teachers. To elaborate further, teachers between the ages of 50 and 64 demonstrate a comparatively diminished degree of AI awareness in comparison to other demographic groups. In the study by Yıldırımer and Karataş (2025), which examined teachers’ attitudes toward AI, it was concluded that the propensity to adopt AI in educational activities decreases with age and seniority. A review of the extant literature reveals a divergence of opinion regarding the impact of factors such as professional seniority, age, and professional experience on AI awareness. While some studies suggest that these factors do not significantly influence AI awareness, others report a negative correlation between age and AI awareness, as well as between age and attitude toward AI. It can be argued that younger teachers may have higher AI awareness than older teachers due to their faster adaptation to technology. Conversely, the present study found that seniority does not have a significant effect on AI awareness. This suggests that the level of AI awareness may not be solely contingent on factors such as seniority or experience. It is plausible that the phenomenon may be associated with the personal initiatives, requisites, and inclination of teachers to be receptive to novel approaches. It is recommended that this variable be re-tested in subsequent studies to further investigate its role in the context of the study. This is due to the belief that professional seniority may serve as an effective factor in the widespread use of AI training and implementation.

This study also found that the level of schools (primary school, middle school, and high school) at which teachers work does not have a significant effect on their AI awareness levels. This finding aligns with the results of studies conducted by Ünal (2025) and Güneyli et al. (2024). The findings of both studies indicate that the level of schools does not exert a significant influence on the AI awareness levels of teachers. Consequently, it can be posited that there is no substantial discrepancy in teachers’ AI awareness levels based on the educational institution they are employed by. Although AI technologies have gained considerable momentum in recent years, the diversity of these tools, their high costs, and the need for advanced infrastructure make large-scale integration into educational contexts challenging. Moreover, there are several drawbacks associated with this approach. Primarily, the field of AI is in its nascent stages of development. It has yet to be incorporated into educational curricula to a significant extent, and educational institutions lack the necessary resources to facilitate its integration. Moreover, the exclusion of AI-focused programs from the professional development of teachers has constrained their knowledge and skills in this domain. Consequently, it can be posited that there is no substantial discrepancy in the awareness levels of teachers working at disparate levels of schools.

The further findings of this study demonstrate that teachers who have undergone training in AI exhibit higher levels of awareness compared to those who have not received such training. Furthermore, it has been observed that teachers who engage with publications concerning technological advancements exhibit a heightened level of AI awareness in comparison to those who do not partake in such professional reading. Receiving training on AI and reading publications with technology content has been shown to increase AI awareness. As highlighted in a previous study, unfamiliarity with technological tools led to notable difficulties among teachers during initial use. Importantly, the same study also revealed that teachers who had received technology training exhibited significantly higher self-efficacy scores regarding technology integration compared to those who had not received such training (Kaymak and Titrek, 2021). According to Banaz and Demirel (2024), reading publications on AI has been identified as a factor that contributes to an increased level of AI literacy. In a separate study, a moderately positive relationship was identified between digital competencies and AI awareness (Gaber et al., 2023). The findings indicate that in-service training can increase AI awareness. It is hypothesized that increasing teachers’ digital literacy through reading publications on AI will positively impact AI awareness. Consequently, it is recommended that courses incorporating AI content be incorporated into the curricula of pre-service teachers. Furthermore, it is imperative that in-service teachers be provided with targeted training activities to facilitate their integration of AI into their pedagogical practices.

In the preceding discussions, the quantitative results regarding the level of AI awareness and the various factors affecting it have been reviewed. In order to comprehend the perception of the AI concept by the participants, their conceptual understanding, and more in-depth individual evaluations regarding the areas of AI use and its effects, the results obtained from 49 teachers are discussed below. These qualitative findings are evaluated and presented as complementary to the quantitative results. The following findings are derived from the qualitative results obtained within the scope of the study.

Forty-one out of forty-nine teachers were observed presenting definitions of the AI concept in this study. In these definitions, AI is expressed as machine learning, technology that facilitates daily life, systems that emulate human behavior, educational tools, data analysis software, and automation tools. While a few teachers defined AI as a tool that carries risk and danger potential, some teachers offered critical evaluations, stating that AI could negatively affect innovation and encourage complacency. This conclusion is further substantiated by the existence of preliminary findings that align with the study’s results. In a metaphorical study conducted by Gölbaşı and Okul (2024), it was found that pre-service teachers evaluated AI as a tool that facilitates access to information and research and saves time but also carries potential danger. A study conducted with teachers and school administrators revealed that a multitude of metaphors are employed to describe AI, including robot, artificial human, computer and machine. The study also determined that the participants lacked sufficient and accurate information about AI (Aktaş, 2021). The findings of the study by Küçükkara et al. (2024) are consistent with the present findings. In the study, educators indicated that AI is an effective tool for reducing the time spent on tasks. Besides, the present study also found that some participants evaluated the effects of AI instead of defining it or did not express an opinion about the AI concept. Consequently, while the majority of participants demonstrated an ability to define AI, a significant proportion did not offer any perspective on the subject or incorporate it into their own views. This situation indicates that, for some teachers, AI awareness remains limited at the level of conceptual knowledge. This qualitative finding serves to explain the quantitative result related to the theoretical knowledge sub-dimension of AI awareness. The quantitative data reveal that awareness in this dimension includes certain limitations. The qualitative findings further clarify that, for some participants, these limitations originate from an underdeveloped conceptual understanding of AI.

A subsequent examination of teachers’ AI awareness regarding its use in general areas reveals a frequent emphasis on economic, health, and education sectors. Areas of emphasis include communication, security, and transportation, though at a moderate level. However, it is evident that the artistic and social domains are expressed in a more constrained manner compared to other domains. Consequently, teachers’ perceptions of the domains of AI use are more focused on practical applications. However, the extent to which it is utilized within social and cultural contexts remains under-explored. In summary, there is a paucity of comprehensive AI awareness regarding the general domains of its application. A more comprehensive awareness training is required, one that includes the versatile areas of AI use.

The findings of this study indicate that AI has the potential to be utilized in a variety of educational settings. While they recognize AI as a significant technological advancement, they also regard it as a multifaceted element that facilitates pedagogical and cognitive processes. The emphasis placed on the utilization of AI in educational settings is predominantly focused on the following domains: individualization of education, effective learning-teaching methodologies, measurement-evaluation processes, concretization of educational concepts, and material development. However, due to their limited AI awareness, they do not mention its use for curriculum development, content creation, or classroom management. These findings are consistent with those reported in previous studies. For instance, in the study conducted by Seyrek et al. (2024), it was determined that AI can be utilized by teachers for tasks such as material preparation and performance evaluation. In a similar vein, the study by Çetin and Yıldız Baklavacı (2024) posits that AI and Industry 4.0 tools can be utilized for a variety of purposes, including the provision of customized learning materials, the enhancement of the learning process, the facilitation of rapid learning, the assurance of permanence, and the support of education and training processes by teachers. Conversely, the observation that more than half of the participants did not mention functions such as ensuring permanent learning, developing curricula, preparing content, and assisting the teacher suggests that their AI awareness is very limited. When this finding is considered together with the limited awareness observed in the ability to associate and belief–attitude sub-dimensions of the scale, it reveals a noteworthy pattern. Teachers are able to associate AI primarily with in-class, micro-level applications. In contrast, awareness of how AI could be integrated into higher-level and more systemic processes such as curriculum development, content design, and classroom management appears to be quite limited. Taken together, these findings suggest that teachers essentially position AI as a tool that supports existing instructional practices rather than as a potential driver of broader pedagogical or systemic change. Consequently, it is recommended that the educational integration of AI be meticulously designed, incorporating comprehensive and applied content within in-service training activities.

A thorough examination of the findings of this study regarding the effects of AI on the education-training process reveals that both positive and negative effects are included in the participant reports. A substantial body of research has emerged to support this finding (Çetin and Yıldız Baklavacı, 2024; Gölbaşı and Okul, 2024; Küçükkara et al., 2024; Seyrek et al., 2024). The positive effects for teachers include individualized instruction, facile access to information, enriching learning environments, time savings, the development of materials, permanent learning, the attraction of attention, and assistance to the teacher. The following are also posited by them as positive effects, albeit with less frequency: experimentation, cost reduction, productivity, and multi-dimensional thinking. On the other hand, the negative effects they highlight include, but are not limited to, the following: decreased motivation, decreased reading of literature, decreased research-thinking skills, decreased preparation, decreased social–emotional connection, decreased privacy and security, decreased addiction, decreased moral problems, increased inequality, decreased employment, decreased motivation, and decreased psychological problems. A substantial body of previous research has emerged to support these findings regarding positive and negative perceptions of AI (Al-Zahrani, 2024; Çetin and Yıldız Baklavacı, 2024; Seyrek et al., 2024; Yim and Wegerif, 2024). These findings indicate that a significant proportion of participants hold contradictory and ambivalent views regarding AI. While the pedagogically supportive aspects of AI—such as personalized instruction, rapid access to information, and the enrichment of the teaching–learning process—are emphasized, concerns related to motivation, critical thinking, social–emotional interaction, and ethical dimensions continue to persist. This situation suggests that teachers’ perspectives on AI are neither entirely optimistic nor entirely negative; rather, they reflect a cautious awareness grounded in a balance between benefits and risks. At the same time, the simultaneous expression of positive and negative evaluations indicates that teachers experience an internal tension with respect to AI technologies.

This situation can be explained within the framework of technology acceptance and the Diffusion of Innovations theory. From the perspective of one of the acceptance models, namely UTAUT, participants acknowledge the pedagogical benefits of AI (performance expectancy), yet remain hesitant due to the inadequacy of facilitating conditions such as training, policy support, and reliability (Nikolic et al., 2024). This creates an ambivalence stemming from the coexistence of perceived benefits and perceived barriers. According to Rogers (1962) Diffusion of Innovations theory, people evaluate an innovation by considering its relative advantage, its compatibility with existing values and practices, and its perceived level of complexity. In this context, while AI offers relative advantages for teachers in terms of personalization, time savings, and efficiency, it also entails uncertainty and complexity due to risks such as decreased motivation, weakened research–thinking skills, weakened social–emotional connections, and privacy and ethical concerns. Therefore, the ambivalence observed can be interpreted as a natural outcome of the simultaneous evaluation of the potential benefits of AI and certain uncertainties associated with innovations.

A comprehensive analysis of teachers’ responses to whether AI can replace the teaching profession reveals that more than half believe it cannot. Generally, the majority of participants who perceive teachers as guides and role models hold the conviction that machines cannot substitute for the human-oriented teaching profession, citing a variety of reasons. The underlying rationale for this phenomenon pertains to the fundamental distinction between the transfer of knowledge, which can be replicated by AI, and the cultivation of emotional competencies such as empathy and bonding, which are inherently human and not yet fully replicable by current technological frameworks. The findings indicate a prevalent concern among participants regarding the significance of emotional development in education, which is comparable to the importance attributed to cognitive skills. It has been documented that educators often perceive AI as deficient in socio-emotional competencies, particularly in the context of providing guidance (Oh and Ahn, 2024). The present finding that AI cannot replace the teaching profession at its current level of development is consistent with the findings of Çetin and Yıldız Baklavacı (2024) and Çetin and Aktaş (2021). This finding aligns with the results of a study by Chan and Tsi (2023), which reported that while a minority of participants expressed concerns about the potential of AI to substitute for educators in the future. However, the majority of participants expressed confidence in the continued necessity of the teaching profession, citing unique human capabilities such as critical thinking, innovation, and emotional intelligence as key factors in education. In the present study, while participants who expressed the opinion that AI could not replace teachers provided thorough and detailed justifications, there were also participants who defended the idea that it could. Consequently, it can be posited that the predominant opinion among participants is that AI is not a substitute for teachers. However, proponents of this technology posit that, when utilized deliberately, it can function as an effective aid for educators.

This study utilizes a mixed methods approach to examine teachers’ AI awareness, presenting comprehensive results that may contribute to the field of AI awareness. The quantitative component of the study presents descriptive and comparative findings concerning their AI awareness. The qualitative findings and results of the study are then used to map teachers’ strengths and weaknesses regarding AI. In this regard, it has been observed that their AI awareness is at a medium level. Consequently, the level of satisfaction is not commensurate with the desired level. Conversely, their AI awareness can be considered reasonable due to its recent integration into both daily and academic life. As we are in the nascent stages of technological development in the field of AI, there is considerable progress yet to be made. Conversely, for this process to be as efficient and pleasant as possible, it is essential to be cognizant of its strengths and limitations. In this regard, teachers who function as guides in the education-training process must possess the requisite awareness to integrate AI into education. While the study indicates that teachers generally hold a favorable attitude toward AI and possess a reasonable degree of knowledge about its application at present, it also reveals that they lack sufficient awareness regarding certain AI-related issues.

The findings indicate that a substantial proportion of teachers are aware of both the potential benefits of artificial intelligence and the serious risks associated with this technology. This situation is positive in that it demonstrates teachers’ consideration of AI in terms of both its benefits and risks. However, views that focus predominantly on the negative consequences of AI may adversely affect the adoption and use of these technologies for educational purposes. In this respect, the Technology Acceptance Model (TAM) and UTAUT emphasize the determining role of perceived usefulness in individuals’ technology adoption processes (Davis, 1989; Venkatesh et al., 2003). According to Diffusion of Innovations theory, the relative advantage offered by a technology may manifest in different forms, including economic benefits, increased ease of use, and superior functionality. The perceived relative advantage influences the rate at which a technology is adopted (Rogers, 1995). Within this framework, a study examining the adoption of ChatGPT in the United Kingdom found that perceived usefulness was the strongest predictor of attitude toward use and behavioral intention (Durmaz et al., 2025). Similarly, another study reported that perceived usefulness was the most important determinant of the technology adoption process, followed by trust (Othman et al., 2025). In this context, it is recommended that specialized AI tools with pedagogical boundaries be developed in order to reduce the negative effects of AI and enhance its adoption.

Nevertheless, the fact that a small number of teachers expressed exclusively positive or exclusively negative views is noteworthy. This situation indicates that these teachers’ levels of digital literacy may not be sufficient to evaluate AI in a holistic manner. Therefore, it is recommended that digital literacy–focused professional development activities for teachers be strengthened. Viberg et al. (2024) found that teachers with higher AI-EdTech self-efficacy and understanding perceived AI tools as more useful and experienced lower levels of anxiety. Similarly, it has been determined that students with higher digital literacy levels perceive AI tools as more useful and easier to use, and that this perception strengthens their adoption intention (Börekci and Çelik, 2024).

It is important to note that AI systems have the potential to generate false information, exhibit bias, and produce imaginary sources. Furthermore, there is a paucity of research addressing critical concerns such as security, privacy, fraud, and ethical implications. Consequently, it is imperative to educate teachers on the potential challenges that may arise when utilizing AI tools, whether for general purposes or educational objectives. The enhancement of AI awareness regarding its potential risks and hazards can serve as a preventative measure against such threats. Researchers interested in conducting studies in this field may examine the challenges educators face in incorporating AI in their practices. The integration of mixed methods in the design of future studies promises to offer a more nuanced and comprehensive perspective. Furthermore, it is proposed that certain variables previously identified as having minimal impact on AI, such as seniority and level of employed school, be subjected to further examination. This is due to the belief that certain individual and institutional variables may influence the level of awareness as the utilization of AI in education becomes pervasive.

As is the case in other studies in the social sciences, this study is not without its limitations. The participants of the study consist of teachers in a specific city. The external validity of the findings may be constrained due to the potential for variation in the characteristics of teaching professionals across different geographical regions. The data collected in the study are limited to the items included in the data collection instruments. Moreover, as the data are based on teachers’ self-reports, there is a potential for self-report bias and social desirability effects. As the results of the study pertain to a phenomenon that is still in the development phase, it is possible that future studies of a similar nature may yield different results.

## Data Availability

The original contributions presented in the study are included in the article/Supplementary material, further inquiries can be directed to the corresponding author.
