# Correction: Mapping teachers' awareness of artificial intelligence in the changing education paradigm: insights from a mixed methods inquiry

**DOI:** 10.3389/fpsyg.2026.1803378

**Published:** 2026-03-03

**Authors:** Ramazan Bulut

**Affiliations:** Faculty of Education, Afyon Kocatepe University, Afyonkarahisar, Türkiye

**Keywords:** AI ethics, AI risks and benefits, artificial intelligence, digitalization, education, teacher awareness

Triantafyllou (2024) was not cited in the article. The citation has now been inserted in the Introduction section, first paragraph, and should read:

“The capacity of AI to emulate human decision-making, learning, and problem-solving skills (Triantafyllou, 2024) is a subject that can be debated and discussed.”

The original version of this article has been updated.

