# Supplementary material for: Mapping teachers’ awareness of artificial intelligence in the changing education paradigm: insights from a mixed methods inquiry
Source: Front Psychol. 2026 Jan 29;17:1687155. doi: 10.3389/fpsyg.2026.1687155 (PMC12894269; doi:10.3389/fpsyg.2026.1687155)
Supplement: Supplementary file 1 [file Supplementary_file_1.docx]

Supplementary Material

# Supplementary Figures


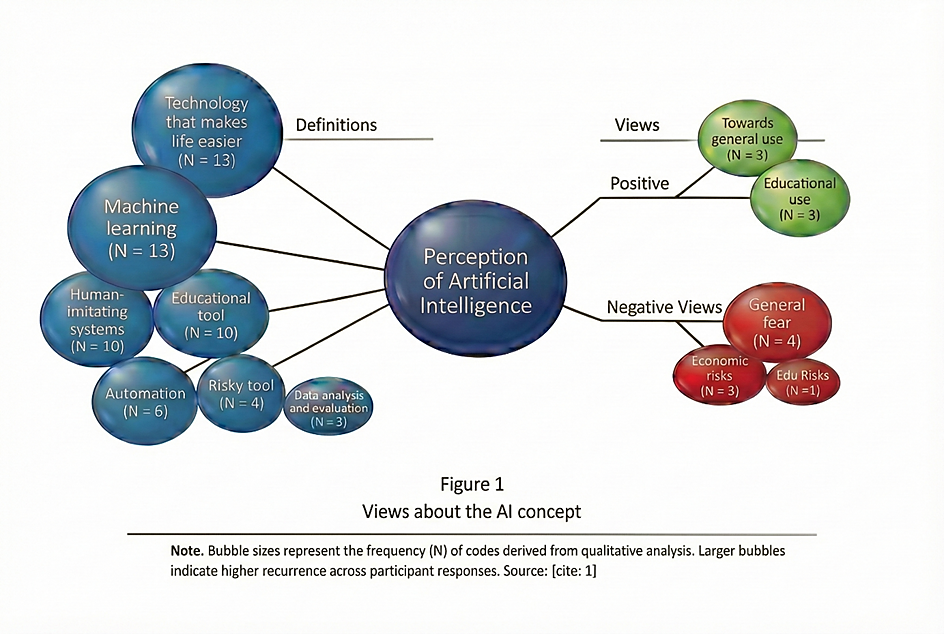


**Supplementary Figure 1.** Views About the Concept of AI


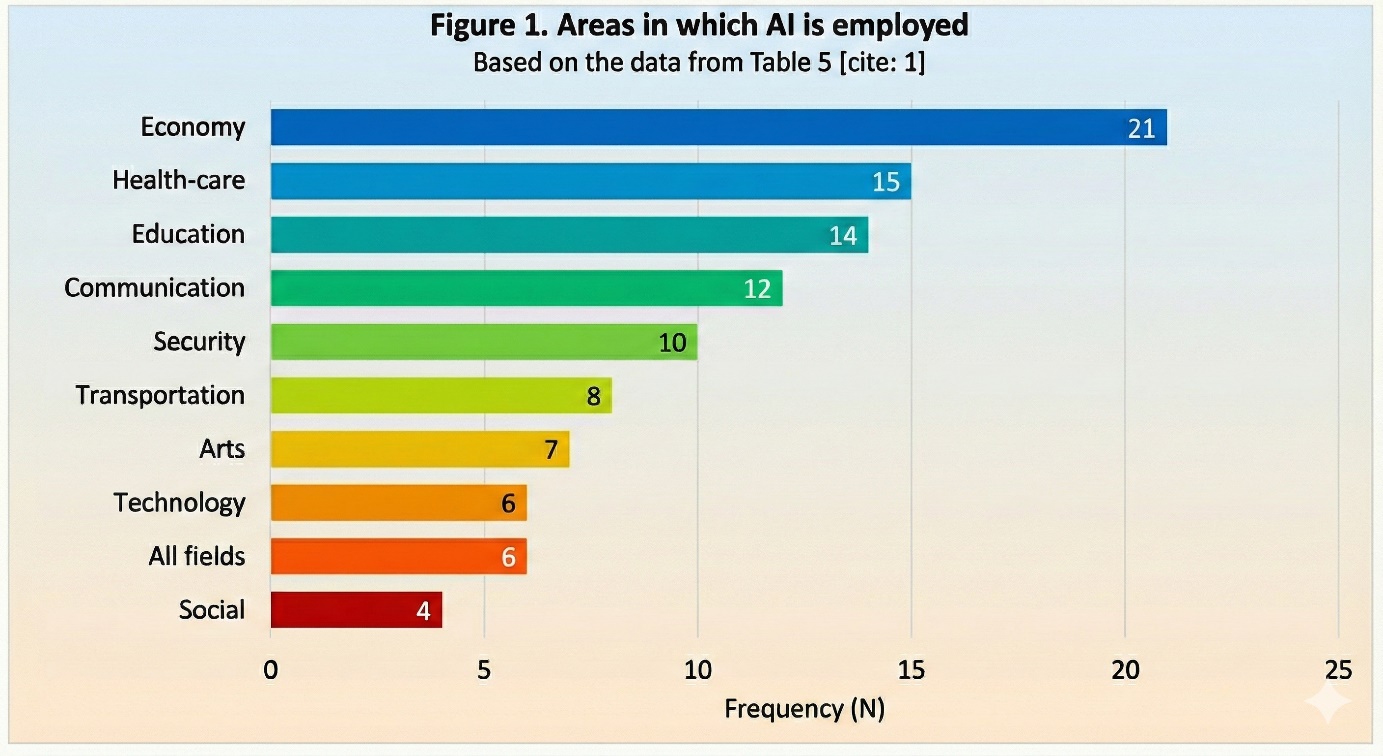


**Supplementary Figure 2.** Areas in which AI is employed


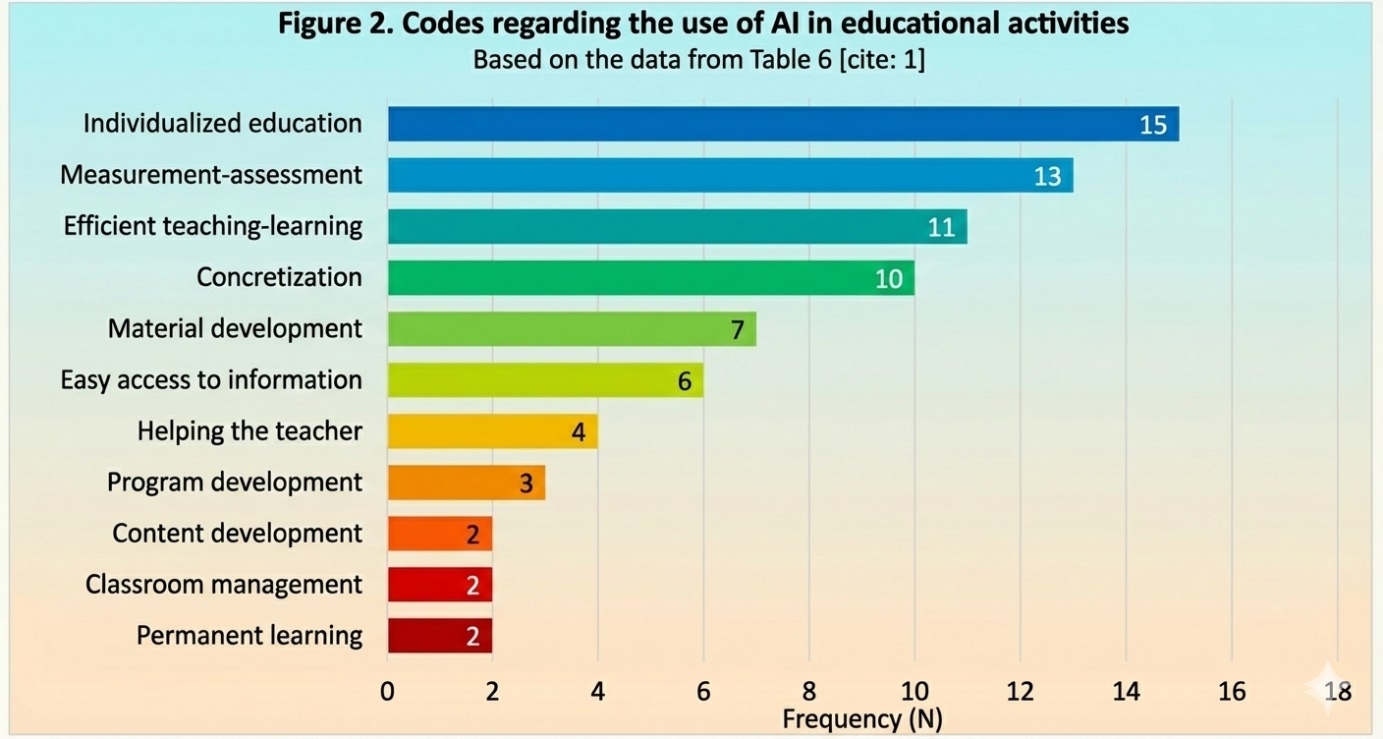


**Supplementary Figure 3.** Educational Areas of AI Use According to Teachers’ Views


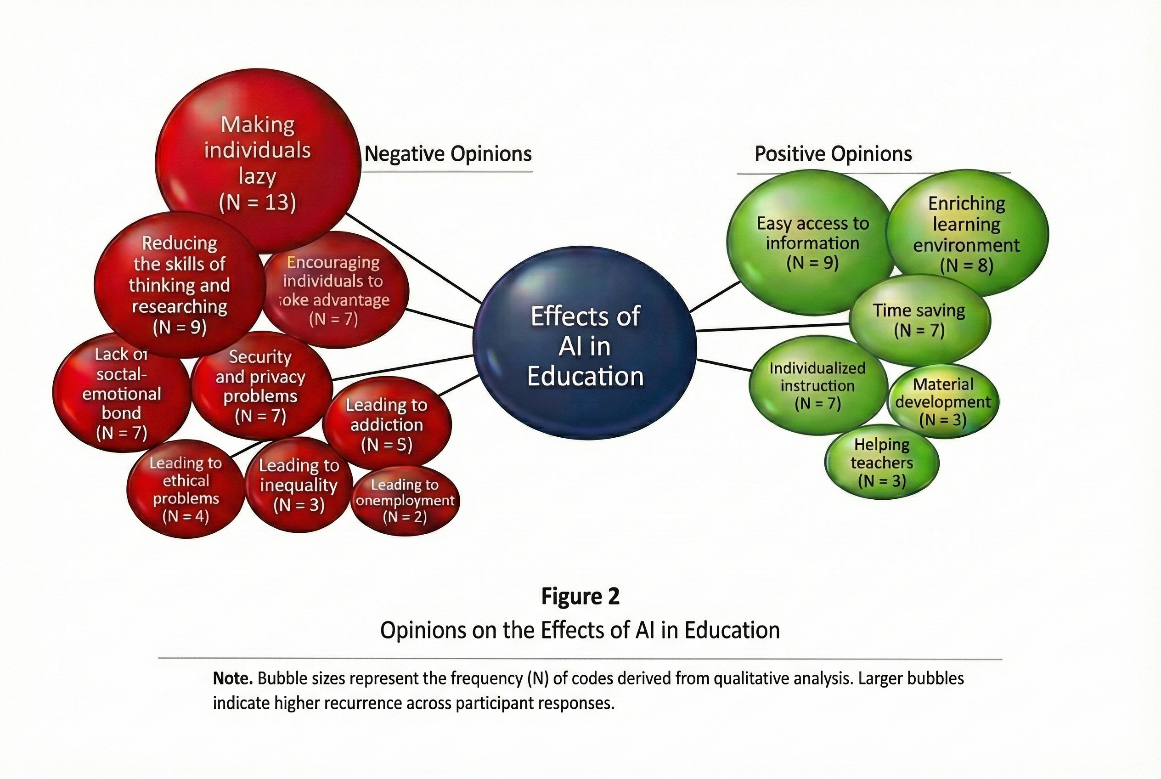
**Supplementary Figure 4.** Negative and Positive Opinions on the Effects of AI in Education

**Supplementary Figure 5.** Views on the impact of AI on the teaching profession.
